# A field‐capable rapid plant DNA extraction protocol using microneedle patches for botanical surveying and monitoring

**DOI:** 10.1002/aps3.11529

**Published:** 2023-06-14

**Authors:** Jonathan Selz, Nicolas R. Adam, Céline E. M. Magrini, Fulvia Malvido Montandon, Sven Buerki, Sebastian J. Maerkl

**Affiliations:** ^1^ Institute of Bioengineering, School of Engineering École Polytechnique Fédérale de Lausanne (EPFL) Station 17 CH‐1015 Lausanne Switzerland; ^2^ Department of Biological Sciences Boise State University 1910 University Drive Boise Idaho 83725 USA

**Keywords:** DNA extraction, field, microneedles, plant, rapid, sequencing

## Abstract

**Premise:**

A novel protocol for rapid plant DNA extraction using microneedles is proposed, which supports botanic surveys, taxonomy, and systematics. This protocol can be conducted in the field with limited laboratory skills and equipment. The protocol is validated by sequencing and comparing the results with QIAGEN spin‐column DNA extractions using BLAST analyses.

**Methods and Results:**

Two sets of DNA extractions were conducted on 13 species spanning various leaf anatomies and phylogenetic lineages: (i) fresh leaves were punched with custom polymeric microneedle patches to recover genomic DNA, or (ii) QIAGEN DNA extractions. Three plastid (*matK*, *rbcL*, and *trnH‐psbA*) and one nuclear ribosomal (ITS) DNA regions were amplified and sequenced using Sanger or nanopore technology. The proposed method reduced the extraction time to 1 min and yielded the same DNA sequences as the QIAGEN extractions.

**Conclusions:**

Our drastically faster and simpler method is compatible with nanopore sequencing and is suitable for multiple applications, including high‐throughput DNA‐based species identifications and monitoring.

The increasing human population puts ever‐growing pressure on natural ecosystems through deforestation, natural resource exploitation, and climate change. Earth's biodiversity is disappearing at an alarming rate, placing ecosystems and their services in jeopardy (Antonelli et al., 2020). These services are crucial to support life as we know it, including factors such as food production, medicinal resources, and materials, as well as the regulation of climate, the formation of fertile soil, and maintaining water and air quality. The protection of nature is crucial to retain its economical and societal benefits (Almond et al., 2020). Biodiversity identification and monitoring are the cornerstones for effective preservation strategies; however, the speed of ecosystem degradation has outpaced the amount of time required for classical identification approaches, with species disappearing faster than they are being identified. To tip the scales of this race against time, initiatives have been put in place to improve the speed of biodiversity assessment (Conservation International, 2022) and to create a database of standardized DNA barcodes for faster species identification (International Barcode of Life, 2022). These DNA barcodes are produced by extracting DNA using well‐established protocols, such as cetyltrimethylammonium bromide (CTAB)‐based or spin‐column extractions, and amplifying the targeted DNA regions using PCR. Sequencing techniques, such as Sanger, are then used to generate barcoding data from these amplicons. This lengthy process relies heavily on laboratory equipment and specialized skills. Next‐generation sequencing (NGS) brought promising prospects for DNA‐based species identification (Buerki and Baker, 2016), and improvements in sequencing technologies facilitated on‐site species identification in remote environments using a mobile laboratory (Pomerantz et al., 2018). However, these genomic methods require lengthy and cumbersome DNA extraction and sample preparation processes, particularly for plants, which impedes efficient field protocols.

Polymeric microneedle (MN) patches were initially developed for drug delivery (Larrañeta et al., 2016) and point‐of‐care diagnostics (He et al., 2020). Recent studies have shown MN patches to be a promising method for rapid DNA extraction, with applications for the detection of allergens in food matrices (Li et al., 2021) and plant pathogen detection (Paul et al., 2019). The MN patch is used to perforate the tissue, which disrupts the surrounding cell walls. The polymer composing the MN patch adsorbs water, concentrating DNA and other molecules on its surface, and the DNA can then be recovered by rinsing the patch with an elution buffer or water. These DNA extractions using MN patches require virtually no equipment or laboratory skills and take less than a minute to execute. In addition, this non‐destructive method does not require large amounts of plant tissue or lengthy processing steps, such as grinding, and is therefore a promising option for fragile or scarce materials such as herbarium samples. However, quantifications of MN extractions revealed lower DNA concentrations and purities than extractions obtained using methods such as CTAB (Paul et al., 2019). Furthermore, it is still unclear whether MN DNA extractions can be used to extract plant genomic DNA, particularly nuclear DNA. If proven suitable for plant DNA extractions, this technique could be used to support rapid botanic surveys as well as taxonomic and systematics studies by reducing the time, cost, and skill barriers associated with genomic DNA extraction methods. Here, we propose a new protocol to test this approach and compare the results with spin‐column DNA extractions.

In this study, MN patches were shown to be able to extract nuclear DNA from plant samples for species identification using multi‐locus plastid and nuclear DNA barcoding. The proposed protocol (Appendix 1) was developed by the GenoRobotics initiative, an interdisciplinary project of the École Polytechnique Fédérale de Lausanne (EPFL) involving students, engineers, and researchers, and tested on plants from the EPFL campus. The objectives of this study are (1) to develop a custom fabrication process for MN patches using standard laboratory equipment, (2) to develop a rapid and field‐deployable DNA extraction protocol using MN patches, and (3) to sequence not only plastid but also nuclear DNA regions from MN DNA extractions. To achieve these objectives, a cost‐efficient alternative to commercially available MN molds, such as those offered by Blueacre Technology (Dundalk, Ireland), was investigated to reduce the cost of producing the master molds. Furthermore, the quality of MN DNA extraction must enable downstream PCR amplification and Sanger sequencing or NGS using nanopore technology. As the purity of MN DNA extractions was previously shown to be limited, a DNA purification step was added in order to ensure the necessary level of quality. We then employed a multi‐locus approach with the widely used plastid markers *matK*, *rbcL*, and *trnH‐psbA* and the nuclear ribosomal ITS region (including ITS1, 5.8S, and ITS2) (Hollingsworth, 2011). We also applied the same protocols to DNA extracted using a standard spin column–based method. The sequencing results from both DNA extraction methods were compared using BLAST similarity analyses (Morgulis et al., 2008). These sequencing results were then matched against the National Center for Biotechnology Information (NCBI) database to confirm that the correct DNA regions were sequenced, and that species identifications were consistent. Finally, the applicability of the MN extractions to nanopore sequencing was evaluated for two species.

## METHODS AND RESULTS

### Materials

**Figure 1:**
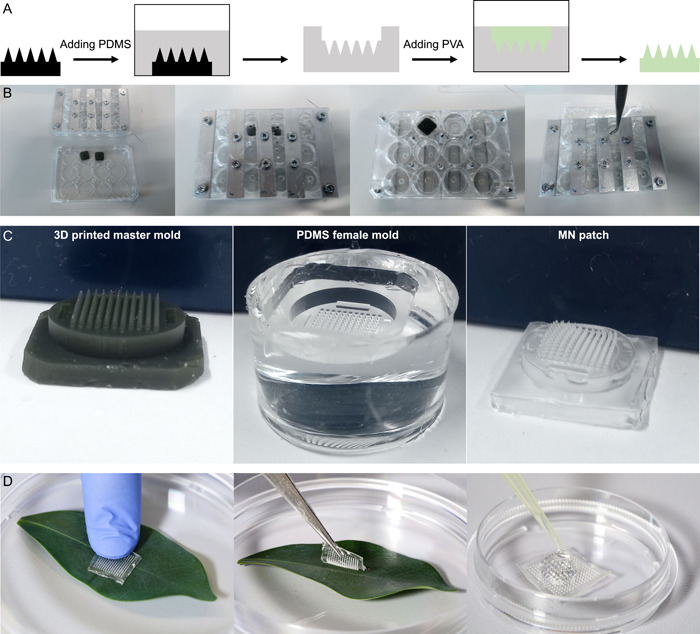
Microneedle (MN) patch fabrication and usage. (A) Schematic of the fabrication steps from the 3D‐printed master mold to the final polymeric MN patch. (B) Main steps to cast polydimethylsiloxane (PDMS) molds using the master molds and a modified 12‐well plate. (C) The three main steps in the MN fabrication process. (D) Demonstration of the DNA extraction process consisting of punching a leaf sample with the MN patch and eluting the DNA.

Custom MN patches were produced in a three‐step process (Figure 1A–C): printing master molds, using the master molds to create polydimethylsiloxane (PDMS) molds, and casting MN patches using the PDMS molds (Wang et al., 2017). The MN patch is made of poly(vinyl alcohol) hydrogel (PVA) and comprises a 1‐cm^2^ array of 121 conical needles with a height of 1600 µm. The master molds were 3D printed by the Atelier de Fabrication Additive at EPFL in HTM 140 resin using the Perfactory 4 Mini XL 3D printer (EnvisionTec, Dearborn, Michigan, USA) using SLA/DLP technology, which has a resolution in the order of tens of microns. The master molds were then used to cast PDMS molds with a 1:10 mix of hardener to elastomer of Sylgard 184 (Suter Kunststoffe, Fraubrunnen, Switzerland). A solution of H_2_O (UltraPure DNase/RNase‐Free Distilled Water; Thermo Fisher Scientific, Waltham, Massachusetts, USA) and PVA (Merck & Cie, Schaffhausen, Switzerland) with a 7:1 weight ratio was prepared. The PDMS molds were cleaned in a heated bath of double‐distilled water, allowed to dry, and placed in 12‐well plates; 750 µL of the PVA solution was then added to each well. The plates were then centrifuged at 2900 × *g* (4000 rpm) for 20 min at 40°C. Another 200–750 µL of the PVA solution was added to fill the molds, taking care not to overfill them. After a drying time of 36 h in a fume hood, the MN patches were unmolded and ready for use.

Initially, *Solanum lycopersicum* L. was used as a model to design and test the DNA extraction and amplification protocols, as well as to test the MN fabrication process. In a second phase, the protocol was tested on additional plants to validate its applicability to different phylogenetic lineages. Eight species were sampled around the EPFL campus (46°31′11.050″N, 6°33′57.966″E), and five species were purchased and grown in the laboratory. All selected plants are angiosperms, and all were eudicots except for one specimen of the order Poales, which belongs to the monocots. The selection of samples for this study was based on targeting a wide diversity of plants to test the MN patches on different leaf morphologies. The notable variations are leaf thickness, succulence, and fibrousness; presence and thickness of cuticle; and the presence of latex, oils, and mucilage. The availability of a reference sequence was also a critical selection criterion for the sampled species. The plant samples and their taxonomy are summarized in Appendix 2.

### Microneedle DNA extractions

Genomic DNA was extracted from 2–3 fresh young leaves (Appendix 2), which were stacked to ensure a sufficient thickness for the length of the MNs. A MN patch was then used to puncture the leaves by placing it in an area with as few veins as possible and pressing the patch forcefully against the leaf between the thumb and index finger for 10–15 s. After removing the MN patch from the leaf, the presence of puncture marks was checked visually. DNA was then recovered by eluting the samples from the MN patches with 50 µL of H_2_O (UltraPure DNase/RNase‐Free Distilled Water; Thermo Fisher Scientific). This critical step requires wetting of the full surface of the MN patch by pipetting 50 µL of H_2_O up and down until bubbles form. It is important not to let the water sit for more than 30 s to avoid the dissolution of the MN patch surface. The full extraction process (Figure 1D) requires about 1 min to complete. All MN extractions were then purified with DNA purification columns according to the manufacturer's protocol (QIAquick; QIAGEN, Hilden, Germany), using 50 µL of extracted DNA as an input. The final elution step was performed with 30 µL of H_2_O.

### QIAGEN DNA extractions

DNA extraction with spin columns (DNeasy Plant Mini Kit; QIAGEN) was selected as the reference method for its relative simplicity, speed, reliability, and widespread usage. As DNA extractions from fresh or silica‐dried samples were shown to yield similar quantities of DNA (Chase and Hills, 1991), dried tissue was chosen for the ease of grinding. The leaves were sampled and dried in silica gel for 36 h, after which the leaf tissue was finely ground using a mortar and pestle. The dried leaf powder (20 mg) was used as an input for the DNA extraction protocol, which was carried out according to the manufacturer's instructions. The final elution was performed in two steps of 50 µL and pooled together.

### DNA amplification and sequencing using both DNA extraction methods

Four commonly used plant DNA barcoding regions (Hollingsworth, 2011) were selected: the coding regions of the *matK* and *rbcL* plastid genes (*matK* and *rbcL*), the *trnH*‐*psbA* plastid intergenic spacer, and the ITS region of nuclear ribosomal DNA. The primers used (Appendix 3) were taken from the following sources: matK472F and matK1248R (Yu et al., 2011), rbcLa‐F (Kress and Erickson, 2007) and rbcL724R (Fay et al., 1997), trnH (Tate, 2002) and psbA (Sang et al., 1997), and ITS‐p5 and ITS‐p4 (Cheng et al., 2016). Amplifications of these four target DNA regions were carried out in 50‐µL reactions, containing 25 µL of 3 mM MgCl_2_ PCR master mix (*Taq* 2X Master Mix; New England Biolabs, Ipswich, Massachusetts, USA), 1 µL of each primer (10 µM stock concentration), and either 16 µL of H_2_O and 7 µL of DNA template from the MN extractions or 21 µL of H_2_O and 2 µL of DNA template from the QIAGEN extractions. This volume variation reflects the typical difference in DNA concentration between both extraction methods. The following PCR profile was used: initial denaturation at 95°C for 2 min, followed by 45 cycles of denaturation at 95°C for 30 s, annealing at 54°C for 30 s, and extension at 68°C for 1 min, followed by a final extension at 68°C for 10 min. The PCR products were then sent to a sequencing service (Microsynth, Balgach, Switzerland) for purification and Sanger sequencing of the forward and reverse sequences.

In addition to Sanger sequencing, the applicability of DNA extracted from MN patches to nanopore sequencing was tested. PCR products for the four targeted DNA regions of two species, *Solanum lycopersicum* and *Ficus benjamina* L., were sequenced using a MinION Mk1B (Oxford Nanopore Technologies [ONT], Oxford, United Kingdom). Oxford Nanopore Technologies offers two types of flow cells for the MinION. The Spot‐ON Flow Cell Mk 1 R9 Version 1 (FLO‐MIN106D) has higher sequencing capabilities than the Flongle Flow Cell (FLO‐FLG001) and can therefore generate more sequencing reads, but is 10 times more expensive. The *S. lycopersicum* samples were sequenced using the MinION and a Spot‐ON Flow Cell. The *F. benjamina* samples were sequenced using a Flongle Flow Cell with a Flongle adapter for MinION (ADP‐FLG001) to further reduce the cost of the process. The library preparation of all samples was carried out with ONT's Rapid Barcoding Kit (SQK‐RBK004). The base calling was performed with ONT's MinKNOW software, which generates Fast5 and FASTQ files containing all the sequencing reads.

### Analyses

**Figure 2:**
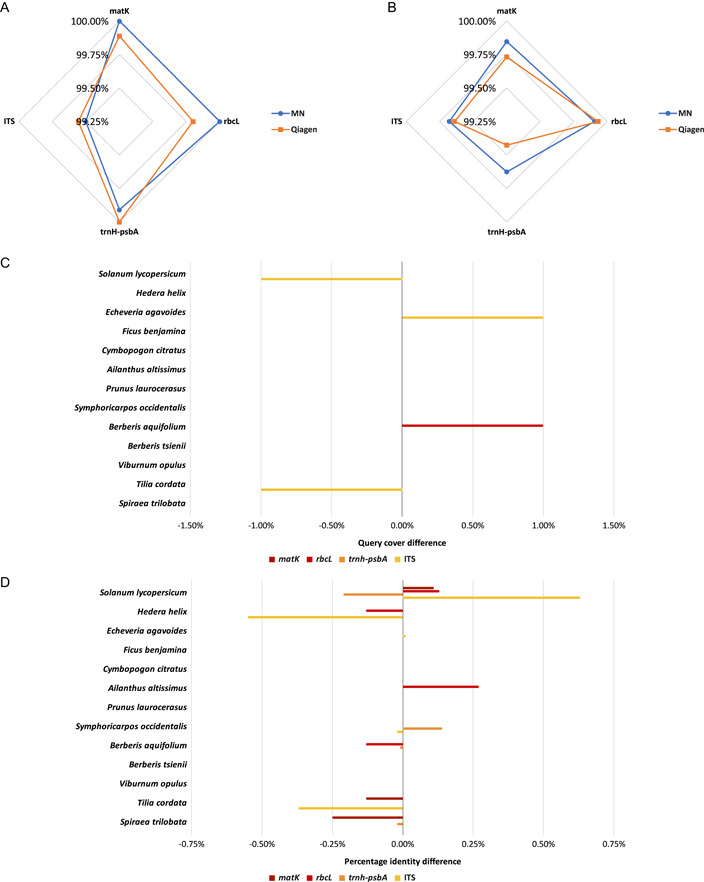
Microneedle (MN) and QIAGEN sequence BLAST alignment results for the four DNA regions sequenced in the 13 plant samples. (A) Average query cover for the alignments of the different DNA regions against the National Center for Biotechnology Information (NCBI) database. (B) Average percentage identity for the alignments of the different DNA regions against the NCBI database. (C) Query cover difference obtained by the subtraction of QIAGEN from the MN results. (D) Percentage identity difference obtained by the subtraction of QIAGEN from the MN results.

To assess the validity of the MN DNA extractions, we compared the sequencing results using the MN‐extracted samples with the sequencing results from DNA samples extracted using the QIAGEN method. The sequencing output composed of forward and reverse sequences was aligned using MAFFT v7 (Katoh and Standley, 2013) on the ab1 files with a minimum quality level of 20 to obtain the consensus sequence of the amplified DNA region. When the overlap between the forward and reverse sequences was too small, consensus sequences were obtained by manually aligning the fragments with the reference sequence from the NCBI database (Table 1). To ensure that both methods produced the same sequences, an initial validation was performed by aligning each MN sequence to its QIAGEN counterpart using BLAST 2 seq (Zhang et al., 2000). These sequences were then searched on GenBank's Nucleotide database using MegaBLAST (Morgulis et al., 2008) to evaluate whether the quality of the sequences enabled species identification (Table 1, Figure 2). The comparison metrics were the identity percentage (IP), which indicates the similarity between the query and the reference sequence, and the query coverage (QC), which is the percentage of the query length aligned with the reference. The analysis of the sequences obtained using ONT nanopore sequencing was performed by aligning the FASTQ reads with a reference sequence, also obtained from GenBank. The DNA sequence alignments were obtained using FASTQ Custom Alignment in the Epi2me software (Oxford Nanopore Technologies) with a minimum Q‐score of 7.

**Table 1:** Summary of the BLAST alignment of the Sanger sequences against the reference sequences of the National Center for Biotechnology Information (NCBI) GenBank Nucleotide database using DNA extracted with the microneedle (MN) and QIAGEN techniques. All values are percentages. See Appendix 2 for details on taxonomy and sampling.

	BLAST alignment on reference					
Species/barcode region	Sequences from MN extractions		Sequences from QIAGEN extractions			MN vs. QIAGEN
	Query cover	% Identity	Query cover	% Identity	Reference	% Identity
*Solanum lycopersicum*						
*matK*	100	100	100	99.89	KY887587.1	100
*rbcL*	100	100	100	99.87	KY887588.1	100
*trnH‐psbA*	100	99.79	100	100	KY887587.1	100
ITS	99	100	100	99.37	OU640345.1	99.37
*Hedera helix*						
*matK*	100	100	100	100	OK539589.1	100
*rbcL*	100	99.87	100	100	OK539589.1	100
*trnH‐psbA*	100	100	100	100	OK539589.1	100
ITS	100	99.45	100	100	MT276681.1	100
*Echeveria agavoides*						
*matK*	N.A.	N.A.	N.A.	N.A.	MG220496.1	
*rbcL*	100	100	100	100	MG220440.1	100
*trnH‐psbA*	No reference	No reference	No reference	No reference	No reference	100
ITS	99	99.57	98	99.56	MF818300.1	100
*Ficus benjamina*						
*matK*	N.A.	N.A.	100	99.77	NC_053834.1	
*rbcL*	N.S.	N.S.	100	100	NC_053834.1	
*trnH‐psbA*	100	99.79	100	99.79	NC_053834.1	100
ITS	100	100	N.S.	N.S.	JN117620.1	
*Cymbopogon citratus*						
*matK*	N.A.	N.A.	N.A.	N.A.	MK593547.1	
*rbcL*	N.A.	N.A.	N.A.	N.A.	MK593547.1	
*trnH‐psbA*	100	100	100	100	MK593547.1	100
ITS	N.S.	N.S.	N.A.	N.A.	ON685417.1	
*Ailanthus altissimus*						
*matK*	N.A.	N.A.	N.A.	N.A.	NC_037696.1	
*rbcL*	100	100	100	99.73	NC_037696.1	100
*trnH‐psbA*	100	100	N.A.	N.A.	NC_037696.1	
ITS	100	99.36	100	99.36	OX327691.1	100
*Prunus laurocerasus*						
*matK*	N.A.	N.A.	100	99.59	NC_068706.1	
*rbcL*	N.A.	N.A.	100	100	NC_068706.1	
*trnH‐psbA*	100	100	100	100	NC_068706.1	100
ITS	N.A.	N.A.	100	99.67	AF318724.1	
*Symphoricarpos occidentalis*						
*matK*	N.A.	N.A.	99	99.36	MT580002.1	
*rbcL*	N.S.	N.S.	100	100	MT580002.1	
*trnH‐psbA*	100	97.63	100	97.49	MT580002.1	100
ITS	100	99.33	100	99.35	FJ217824.1	100
*Berberis aquifolium*						
*matK*	N.A.	N.A.	100	99.36	NC_066183.1	
*rbcL*	100	99.87	99	100	NC_066183.1	100
*trnH‐psbA*	100	99.81	100	99.82	NC_066183.1	100
ITS	No reference	No reference	No reference	No reference	No reference	100
*Berberis tsienii*						
*matK*	N.A.	N.A.	N.A.	N.A.	NC_067774.1	
*rbcL*	N.A.	N.A.	N.A.	N.A.	NC_067774.1	
*trnH‐psbA*	99	100	N.A.	N.A.	NC_067774.1	
ITS	100	100	N.A.	N.A.	JN012234.1	
*Viburnum opulus*						
*matK*	100	100	100	100	LT996894.1	100
*rbcL*	100	99.6	N.A.	N.A.	LT996894.1	
*trnH‐psbA*	100	100	N.S.	N.S.	LT996894.1	
ITS	100	99.86	100	99.86	MT784073.1	100
*Tilia cordata*						
*matK*	100	99.87	100	100	NC_065062.1	100
*rbcL*	N.A.	N.A.	99	99.73	NC_065062.1	
*trnH‐psbA*	N.A.	N.A.	100	98.85	NC_065062.1	
ITS	99	99.63	100	100	MT735332.1	100
*Spiraea trilobata*						
*matK*	100	99.36	100	99.61	MW822176.1	100
*rbcL*	100	100	100	100	MW822176.1	100
*trnH‐psbA*	100	98.87	100	98.89	MW822176.1	100
ITS	98	99.59	98	99.59	KU321589.1	100

*Note*: N.A. = not amplified; N.S. = not sequenced.

### Results

Of the four targeted DNA regions sequenced for 13 plant species (i.e., a total of 52 DNA sequences), 35 sequences were successfully obtained from the MN DNA extractions vs. 39 from the QIAGEN extractions. Unsuccessful amplifications of the targeted DNA regions were the main cause of failure. The use of non‐specific primers and a single PCR profile are the most probable explanations of unsuccessful amplifications; however, the goal of this standardization was to develop a simplified process for field applicability. Sequences were obtained for 38.5% of MN samples vs. 69.2% of QIAGEN samples using *matK*, for 53.8% of MN samples vs. 76.9% of QIAGEN samples using *rbcL*, for 92.3% of MN samples vs. 76.9% of QIAGEN samples using *trnH‐psbA*, and for 84.6% of MN samples vs. 76.9% of QIAGEN samples using the ITS region. Considering the stochasticity in the total number of sequenced samples, both methods performed similarly. The success rate for *matK* was substantially lower than for the other DNA regions; the amplification of this region seems notably more susceptible to sample purity and will require further optimization (Dunning and Savolainen, 2010).

The BLAST analyses performed on the sequencing data from the MN and QIAGEN extractions revealed a 100% similarity between MN and QIAGEN sequences (Table 1). The MN DNA extraction method can therefore be used to generate the same barcode sequencing data as standard QIAGEN extractions. A notable exception is the ITS sequence for *S. lycopersicum*, for which the QIAGEN sample yielded a low‐quality forward sequence. The BLAST comparison against GenBank's database (Table 1, Figure 2) demonstrated that the generated sequences correspond to the targeted DNA regions of the sampled species. The top hits in the database were the same for both the MN and QIAGEN methods, validating the applicability of MN DNA extractions for species identification. The consistency of the IP for the MN sequences also indicates the high repeatability of this method. It is also interesting to note the high IP and QC of the MN method for the ITS region, showing that nuclear DNA can be obtained reliably using this extraction method. For *F. benjamina*, the plastid DNA regions and the ITS region seem to identify two different *Ficus* species, hence the taxonomy could be validated only at the genus level. The nanopore sequencing results (Table 2) showed an excellent similarity between the QIAGEN and MN methods for both *S. lycopersicum* and *F. benjamina*. The minimal average identity is 95.6% for the MN sequences and 96.3% for the QIAGEN sequences, whereas the minimal average accuracy is higher for MN sequences than QIAGEN sequences at 88.6% and 79.0%, respectively. These results confirm the compatibility of MN DNA extractions with NGS and more specifically nanopore sequencing.

**Table 2:** Summary of the alignment of the nanopore sequences against the reference sequences of the National Center for Biotechnology Information (NCBI) GenBank Nucleotide database using DNA extracted from *Solanum lycopersicum* and *Ficus benjamina* with the microneedle (MN) and QIAGEN techniques. All values are percentages. See Appendix 2 for details on taxonomy and sampling.

	FASTQ custom alignment				
Species/barcode region	Sequences from MN extractions		Sequences from QIAGEN extractions		
	Average accuracy	Average identity	Average accuracy	Average identity	Reference
*Solanum lycopersicum*					
*matK*	91.2	97.1	93.1	97.1	KY887587.1
*rbcL*	91.4	96.8	93.7	96.8	KY887588.1
*trnH‐psbA*	91.2	97.5	93.2	97.5	KY887587.1
ITS	88.6	95.6	83.5	99.6	OU640345.1
*Ficus benjamina*					
*matK*	N.A.	N.A.	93	97.6	NC_053834.1
*rbcL*	92.2	96.9	79	97.2	NC_053834.1
*trnH‐psbA*	92.4	97.6	99	97.5	NC_053834.1
ITS	90	97.5	89.3	96.3	JN117620.1

*Note*: N.A. = not amplified.

Both extraction methods produce similar sequencing data; however, spin column–based and MN‐based DNA extractions differ markedly in the way they are carried out. While the QIAGEN spin column extraction requires a thermal heating/cooling block, a centrifuge, several reagents, and consumables, the MN extraction is performed with only a single MN patch, pipette and tip, 1.5‐mL tube, and H_2_O. The MN extraction is a straightforward process that requires minimal expertise, and the process is therefore cost‐ and time‐effective. Microneedle extractions can be performed in 1 min plus ~10 min for purification, while a QIAGEN extraction requires at least 30 min for sample preparation and 90 min for the extraction protocol, making it more than 10 times longer. In terms of cost, the QIAGEN kit costs about $4.50 USD per sample. The fabrication of one MN patch amounts to less than $0.30 USD and the purification costs $1.30 USD per sample, making it 2.75 times cheaper than the commercially available DNA extraction kit. Further improvements can be made to the purification step if needed. Overall, this new protocol has the potential to minimize the current biodiversity crisis by lowering the cost, time, and skill barriers, as well as the need for laboratory infrastructure, when conducting DNA‐based species identifications in a field setting.

## CONCLUSIONS

We introduced a novel protocol to produce low‐cost MN patches in‐house with standard laboratory equipment using custom 3D‐printed master molds. We tested the DNA extractions using these MN patches on plants grown on campus and cultivated in the laboratory and compared them with QIAGEN extractions of the same specimens. Using this method in combination with a purification step, we managed to amplify and sequence plastid and nuclear DNA regions with Sanger and nanopore technologies. The sequencing results showed equivalent outputs for both the MN and QIAGEN extractions, and confirmed the usability of MN sequences for species identification through comparison with a reference database. This supports the application of the proposed technique for plant biodiversity surveying, monitoring, taxonomy, and systematics.

The next step for a truly field‐capable DNA analysis tool would be to overcome the need for thermal cycling during the amplification step by replacing PCR with an isothermal amplification method, such as recombinase polymerase amplification, and coupling it with nanopore sequencing to analyze the amplicons. Additionally, the requirement for the DNA purification step needs to be further investigated. Eliminating this step would reduce the DNA extraction time even further and drastically improve field capability. Our preliminary investigations indicate a seasonal influence on the amplification output. Successful amplifications of unpurified MN DNA extractions were possible using tissue collected in the spring, but all amplifications of unpurified DNA failed when tissue was collected in the late summer and autumn (unpublished data). Possible solutions could involve fine‐tuning the PCR parameters or adapting the MN patch material by using a blend with a polycationic polymer (Kiang et al., 2004). Further investigations are necessary to validate the impact of plant metabolism on MN DNA extractions and to determine the necessity of a purification step.

This protocol provides a simple and rapid method for sample collection and DNA extraction that can be used in the field as well as in large‐scale studies for botanical surveying and monitoring. The MN DNA extraction method yields plant DNA that can be amplified and sequenced at a field station using nanopore technology (Parker et al., 2017), and is 10 times faster and 2.75 times less expensive than conventional QIAGEN extractions. Compared with other commonly used extraction methods, its simplicity, speed, and limited requirement for lab equipment make it suitable for field DNA analysis, and thus MN DNA extractions could serve as a basis for high‐throughput DNA‐based species identifications and monitoring. Its ease of use combined with its low cost make it a first step toward the contribution of citizen science in DNA extractions, which would enable a high potential for data collection, and at the same time promote biodiversity awareness in local communities.

## AUTHOR CONTRIBUTIONS

C.E.M.M., F.M.M., J.S., and N.R.A. conceived the protocols, performed the experiments, and analyzed the data. C.E.M.M. and F.M.M. designed the experiments. J.S. and N.R.A. reviewed the experimental designs and wrote the manuscript. S.J.M. and S.B. provided guidance and critical reviews. All the authors approved the final version of the manuscript.

## Data Availability

The Sanger sequence data produced in this project are available from the National Center for Biotechnology Information (NCBI) GenBank under the following accessions: *matK* sequences OQ929507–OQ929520, *rbcL* sequences OQ929521–OQ929537, *trnH‐psbA* sequences OQ929538–OQ929559, and ITS sequences OQ910036–OQ910056. Nanopore sequence data are available from NCBI nder BioProject accession no. PRJNA965810, BioSample accession no. SAMN34500108–SAMN34500109, and SRA accession no. SRR24386285–SRR24386289.

## Appendix 1 Required materials and protocol to perform the microneedle patch fabrication, DNA extraction, and amplification steps.

The protocol can be accessed online at: https://www.protocols.io/private/8F324FC92A0A11EDBA1E0A58A9FEAC02

**Equipment**:

- 200‐μL and 1000‐μL pipettes
- Vacuum chamber and vacuum source reaching at least 80 kPa of vacuum
- Oven reaching at least 80°C
- Magnetic stirring hotplate and magnetic rod
- 500‐mL beaker
- 50–100‐mL screw‐cap bottles
- Fume hood
- Centrifuge for plates reaching 2900 × *g*
- Tweezers
- Thermocycler
- 3D‐printed master molds

**Consumables**:

- 12‐well plates
- VWR cover film (VWR, Radnor, Pennsylvania, USA)
- P200 and P1000 pipette tips
- Aluminum foil
- Petri dish
- 1.5‐mL Eppendorf tubes
- 0.2‐mL PCR tubes

**Reagents**:

- Polydimethylsiloxane (PDMS)
- DNase‐free water
- ddH_2_O
- Poly(vinyl alcohol) hydrogel (PVA)
- 70% ethanol
- *Taq* 2X Master Mix
- Primers

### Mold fabrication

1. Drill a 5‐mm‐diameter hole in the bottom of each well of a 12‐well plate.
2. Stick the master molds to the lid of the 12‐well plates using double‐sided tape and close the plate.
3. Prepare the PDMS solution with a 1:10 weight ratio of hardener to base and mix it well. For a 12‐well plate, 5 g of PDMS per mold is required.
4. Cast the PDMS using a syringe or a cut‐off pipette tip through the previously drilled holes. We recommend casting about 2/3 of the wells' height.
5. Place the 12‐well plate in a vacuum chamber until off‐gassing is complete (about 30 min).
6. Cure the PDMS in an oven at 80°C for 30–60 min.
7. Take the molds out of the wells and clean them (steps 19–21).

### PVA solution

8. Prepare a solution of DNase‐free water and PVA at a 7:1 mass ratio. Note that a batch of 24 microneedle (MN) patches typically requires 28 g of water for 4 g of PVA.
  - Pour the desired amount of water in a beaker with a magnetic stirrer, add the corresponding weight of PVA, and cover the beaker with aluminum foil.
  - Place on a magnetic stirring hotplate at 60°C and 300 rpm until the solution is homogenized and clear.
9. Transfer to a screw‐cap bottle.
10. Before use, let the solution settle until the foam from the stirring has disappeared (around 1 h but typically overnight).

### Microneedle fabrication

11. Preheat the centrifuge to 40°C.
12. Place the PDMS molds in the 12‐well plates and tape the bottom holes with VWR cover film.
13. Pipette 750 μL of the PVA solution into each mold and then close the well plates.
14. Centrifuge the plates at 4000 rpm and 40°C for 20 min.
15. Pipette enough PVA into each mold (maximum 750 μL) to fill the imprints. Note that overfilling the molds will lead to much longer drying times.
16. Place the plates without a lid under a fume hood and let dry for 36 h.
17. Use tweezers to extract the MN patches from the molds.
18. Remove the molds from the well plates to clean them.

### Mold cleaning

19. Place the PDMS molds in a beaker with a magnetic stirrer, add ddH_2_O until it covers the molds, and cover the beaker with aluminum foil.
20. Place the beaker on a magnetic stirrer hotplate for 1 h at 200–250 rpm and 80–90°C.
21. Take the molds out, rinse them thoroughly with ethanol, shake off the excess, and place them under a fume hood until dry (about 15 min).

### DNA extraction

22. Puncture the leaf sample with a MN patch:
  - Select and stack 2–3 fresh leaves (young leaves are preferred).
  - Place a MN patch on the leaf in an area with the least veins possible.
  - Press forcefully and evenly on the whole patch to puncture the leaf and maintain pressure for 10–15 s.
  - Remove the MN patch and place it on a clean surface (e.g., Petri dish).
23. Elute the DNA by pipetting DNase‐free water onto the MN patch:
  **Note**: For increased DNA concentration, elute with 50 μL. For increased DNA yield, elute with 100 μL.
  - Pipette the DNase‐free water onto the MNs, taking care to wet the whole surface.
  - Pipette up and down until bubbles form in the water. Avoid leaving the water for too long or the MN patch will dissolve, complicating the recovery of the eluted DNA.
  - Recover the eluate in an Eppendorf tube.

### DNA amplification

24. Prepare a 50‐μL PCR reaction mix:
  **Table:**
  	Volume	
  Reagent	Microneedle extractions	QIAGEN extractions
  *Taq* 2X Master Mix	25 μL	25 μL
  Forward primer	1 μL	1 μL
  Reverse primer	1 μL	1 μL
  Water	16 μL	21 μL
  DNA template	7 μL	2 μL
25. Load the sample in a thermocycler and run the following program:

**Table:**

No. of cycles	Step	Temperature	Time
1	Initial denaturation	95°C	2 min
45	Denaturation	95°C	30 s
	Annealing	54°C	40 s
	Extension	68°C	40 s
1	Final extension	68°C	10 min
1	Hold	4°C	∞

## Appendix 2 Plant samples and their taxonomy.

**Table:**

ID	Species	Family	Order	Sampling location	Geographic coordinates
2	*Hedera helix* L.	Araliaceae	Apiales	EPFL	Cultivated
10	*Symphoricarpos occidentalis* Hook.	Caprifoliaceae	Dipsacales	EPFL	46°31′09.730″N, 6°33′51.321″E
13	*Viburnum opulus* L.	Viburnaceae	Dipsacales	EPFL	46°31′09.810″N, 6°33′55.819″E
15	*Tilia cordata* Mill.	Malvaceae	Malvales	EPFL	46°31′08.250″N, 6°34′00.165″E
6	*Cymbopogon citratus* (DC.) Stapf	Poaceae	Poales	EPFL	Cultivated
11	*Berberis aquifolium* Pursh	Berberidaceae	Ranunculales	EPFL	46°31′09.339″N, 6°33′51.022″E
12	*Berberis tsienii* T. S. Ying	Berberidaceae	Ranunculales	EPFL	46°31′09.341″N, 6°33′50.966″E
4	*Ficus benjamina* L.	Moraceae	Rosales	EPFL	Cultivated
9	*Prunus laurocerasus* L.	Rosaceae	Rosales	EPFL	46°31′12.793″N, 6°33′55.076″E
16	*Spiraea trilobata* L.	Rosaceae	Rosales	EPFL	46°31′11.507″N, 6°33′58.433″E
7	*Ailanthus altissimus* (Mill.) Swingle	Simaroubaceae	Sapindales	EPFL	46°31′11.290″N, 6°33′58.347″E
3	*Echeveria agavoides* Lem.	Crassulaceae	Saxifragales	EPFL	Cultivated
1	*Solanum lycopersicum* L.	Solanaceae	Solanales	EPFL	Cultivated

*Note*: EPFL = École Polytechnique Fédérale de Lausanne, Lausanne, Switzerland.

## Appendix 3 PCR primers used in this study.

**Table:**

Primer	5′–3′ sequence	Source
matK472F	CCC RTY CAT CTG GAA ATC TTG GTT C	Yu et al. (2011)
matK1248R	GCT RTR ATA ATG AGA AAG ATT TCT GC	Yu et al. (2011)
rbcLa‐F	ATG TCA CCA CAA ACA GAG ACT AAA GC	Kress and Erickson (2007)
rbcL724R	TCG CAT GTA CCT GCA GTA GC	Fay et al. (1997)
trnH	CGC GCA TGG TGG ATT CAC AAT CC	Tate (2002)
psbA	GTT ATG CAT GAA CGT AAT GCT	Sang et al. (1997)
ITS‐p5	CCT TAT CAY TTA GAG GAA GGA G	Cheng et al. (2016)
ITS‐p4	CCG CTT AKT GAT ATG CTT AAA	Cheng et al. (2016)

## References

[aps311529-bib-0001] Almond, R. E. A. , M. Grooten , and T. Petersen . 2020. Bending the curve of biodiversity loss. Living Planet Report 2020. WWF, Gland, Switzerland.

[aps311529-bib-0002] Antonelli, A. , C. Fry , R. J. Smith , M. S. J. Simmonds , P. J. Kersey , H. W. Pritchard , M. S. Abbo , et al. 2020. State of the world's plants and fungi 2020. Royal Botanic Gardens, Kew, Richmond, Surrey, United Kingdom.

[aps311529-bib-0003] Buerki, S. , and W. J. Baker . 2016. Collections‐based research in the genomic era. Biological Journal of the Linnean Society 117: 5–10.

[aps311529-bib-0004] Chase, M. W. , and H. H. Hills . 1991. Silica gel: An ideal material for field preservation of leaf samples for DNA studies. Taxon 40: 215–220.

[aps311529-bib-0005] Cheng, T. , C. Xu , L. Lei , C. Li , Y. Zhang , and S. Zhou . 2016. Barcoding the kingdom Plantae: New PCR primers for *ITS* regions of plants with improved universality and specificity. Molecular Ecology Resources 16: 138–149. 26084789 10.1111/1755-0998.12438

[aps311529-bib-0006] Conservation International . 2022. Rapid Assessment Program. Website: https://www.conservation.org/projects/rapid-assessment-program [accessed 15 September 2022].

[aps311529-bib-0007] Dunning, L. T. , and V. Savolainen . 2010. Broad‐scale amplification of *matK* for DNA barcoding plants, a technical note. Botanical Journal of the Linnean Society 164: 1–9.

[aps311529-bib-0008] Fay, M. F. , S. M. Swensen , and M. W. Chase . 1997. Taxonomic affinities of *Medusagyne oppositifolia* (Medusagynaceae). Kew Bulletin 52: 111–120.

[aps311529-bib-0009] He, R. , Y. Niu , Z. Li , A. Li , H. Yang , F. Xu , and F. Li . 2020. A hydrogel microneedle patch for point‐of‐care testing based on skin interstitial fluid. Advanced Healthcare Materials 9: 1901201. 10.1002/adhm.201901201 31957291

[aps311529-bib-0010] Hollingsworth, P. M. 2011. Refining the DNA barcode for land plants. Proceedings of the National Academy of Sciences, USA 108: 19451–19452. 10.1073/pnas.1116812108 PMC3241790 22109553

[aps311529-bib-0011] International Barcode of Life . 2022. DNA barcoding. Website: https://ibol.org/about/dna-barcoding/ [accessed 15 September 2022].

[aps311529-bib-0012] Katoh, K. , and D. M. Standley . 2013. MAFFT Multiple Sequence Alignment Software Version 7: Improvements in performance and usability. Molecular Biology and Evolution 30: 772–780. 23329690 10.1093/molbev/mst010 PMC3603318

[aps311529-bib-0013] Kiang, T. , J. Wen , H. W. Lim , and K. W. Leong . 2004. The effect of the degree of chitosan deacetylation on the efficiency of gene transfection. Biomaterials 25: 5293–5301. 15110480 10.1016/j.biomaterials.2003.12.036

[aps311529-bib-0014] Kress, W. J. , and D. L. Erickson . 2007. A two‐locus global DNA barcode for land plants: The coding *rbcL* gene complements the non‐coding *trnH‐psbA* spacer region. PLoS ONE 2: e508. 17551588 10.1371/journal.pone.0000508 PMC1876818

[aps311529-bib-0015] Larrañeta, E. , R. E. M. Lutton , A. D. Woolfson , and R. F. Donnelly . 2016. Microneedle arrays as transdermal and intradermal drug delivery systems: Materials science, manufacture and commercial development. Materials Science and Engineering: R: Reports 104: 1–32.

[aps311529-bib-0016] Li, H. , J. Feng , Y. Wang , G. Liu , X. Chen , and L. Fu . 2021. Instant and multiple DNA extraction method by microneedle patch for rapid and on‐site detection of food allergen‐encoding genes. Journal of Agricultural and Food Chemistry 69: 6879–6887. 34105975 10.1021/acs.jafc.1c01077

[aps311529-bib-0017] Morgulis, A. , G. Coulouris , Y. Raytselis , T. L. Madden , R. Agarwala , and A. A. Schäffer . 2008. Database indexing for production MegaBLAST searches. Bioinformatics 24: 1757–1764. 18567917 10.1093/bioinformatics/btn322 PMC2696921

[aps311529-bib-0018] Parker, J. , A. J. Helmstetter , D. Devey , T. Wilkinson , and A. S. T. Papadopulos . 2017. Field‐based species identification of closely‐related plants using real‐time nanopore sequencing. Scientific Reports 7: 8345. 28827531 10.1038/s41598-017-08461-5 PMC5566789

[aps311529-bib-0019] Paul, R. , A. C. Saville , J. C. Hansel , Y. Ye , C. Ball , A. Williams , X. Chang , et al. 2019. Extraction of plant DNA by microneedle patch for rapid detection of plant diseases. ACS Nano 13: 6540–6549. 31179687 10.1021/acsnano.9b00193

[aps311529-bib-0020] Pomerantz, A. , N. Peñafiel , A. Arteaga , L. Bustamante , F. Pichardo , L. A. Coloma , C. L. Barrio‐Amorós , et al. 2018. Real‐time DNA barcoding in a rainforest using nanopore sequencing: Opportunities for rapid biodiversity assessments and local capacity building. GigaScience 7: giy033. 29617771 10.1093/gigascience/giy033 PMC5905381

[aps311529-bib-0021] Sang, T. , D. J. Crawford , and T. F. Stuessy . 1997. Chloroplast DNA phylogeny, reticulate evolution, and biogeography of *Paeonia* (Paeoniaceae). American Journal of Botany 84: 1120–1136. 21708667

[aps311529-bib-0022] Tate, J. A. 2002. Systematics and evolution of *Tarasa* Philippi (Malvaceae): An enigmatic Andean polyploid genus. PhD dissertation, University of Texas, Austin, Texas, USA.

[aps311529-bib-0023] Wang, M. , L. Hu , and C. Xu . 2017. Recent advances in the design of polymeric microneedles for transdermal drug delivery and biosensing. Lab on a Chip 17: 1373–1387. 28352876 10.1039/c7lc00016b

[aps311529-bib-0024] Yu, J. , J.‐H. Xue , and S.‐L. Zhou . 2011. New universal *matK* primers for DNA barcoding angiosperms. Journal of Systematics and Evolution 49: 176–181.

[aps311529-bib-0025] Zhang, Z. , S. Schwartz , L. Wagner , and W. Miller . 2000. A greedy algorithm for aligning DNA sequences. Journal of Computational Biology 7: 203–214. 10890397 10.1089/10665270050081478

